# Coexisting Multiple Charge Orders and Magnetism in the Kagome Superconductor LaRu_3_Si_2_

**DOI:** 10.1002/adma.202503065

**Published:** 2025-07-23

**Authors:** C. Mielke, V. Sazgari, I. Plokhikh, Mingsheng Yi, S. Shin, H. Nakamura, J.N. Graham, J. Küspert, I. Biało, G. Garbarino, D. Das, M. Medarde, M. Bartkowiak, J.‐X. Yin, S.S. Islam, R. Khasanov, H. Luetkens, M.Z. Hasan, E. Pomjakushina, M. H. Fischer, J. Chang, T. Neupert, S. Nakatsuji, B. Wehinger, Gang Xu, D.J. Gawryluk, Z. Guguchia

**Affiliations:** ^1^ PSI Center for Neutron and Muon Sciences CNM Villigen PSI 5232 Switzerland; ^2^ Physik‐Institut Universität Zürich Winterthurerstrasse 190 Zürich CH‐8057 Switzerland; ^3^ Wuhan National High Magnetic Field Center and School of Physics Huazhong University of Science and Technology Wuhan 430074 China; ^4^ Institute for Solid State Physics (ISSP) University of Tokyo Kashiwa Chiba 277‐8581 Japan; ^5^ Department of Physics Southern University of Science and Technology Shenzhen Guangdong 518055 China; ^6^ Laboratory for Topological Quantum Matter and Advanced Spectroscopy (B7) Department of Physics Princeton University Princeton New Jersey 08544 USA; ^7^ European Synchrotron Radiation Facility 71 Avenue des Martyrs Grenoble 38000 France

**Keywords:** charge order, Kagome lattice, muon‐spin rotation, superconductivity, time‐reversal symmetry‐breaking

## Abstract

The Kagome lattice has emerged as a promising platform for hosting unconventional chiral charge orders at high temperatures. In the context of the correlated Kagome superconductor LaRu_3_Si_2_, a room‐temperature charge‐ordered state with a propagation vector of ($\frac{1}{4}$, 0, 0) is previously reported. However, understanding the interplay between this charge order and superconductivity, particularly with respect to time‐reversal‐symmetry breaking, remains elusive. In this study, we employ single‐crystal X‐ray diffraction, magnetotransport measurements, muon‐spin rotation experiments, and first‐principles calculations to investigate charge order and its electronic and magnetic responses in LaRu_3_Si_2_ across a wide temperature range, down to the superconducting state. These findings reveal the appearance of a charge order with a propagation vector of ($\frac{1}{6}$, 0, 0) below *T*_co, 2_ ≃ 80 K, which coexists with the previously identified room temperature primary charge order ($\frac{1}{4}$, 0, 0). The primary charge‐ordered state exhibits zero magnetoresistance. In contrast, the appearance of the secondary charge order at *T*_co, 2_ is accompanied by a notable magnetoresistance response and a pronounced temperature‐dependent Hall effect, which experiences a sign reversal, switching from positive to negative below T* ≃ 35 K. Intriguingly, a significant enhancement is observed in the internal field width sensed by the muon ensemble below T* ≃ 35 K. Moreover, the muon spin relaxation rate exhibits a substantial increase upon the application of an external magnetic field below *T*_co, 2_ ≃ 80 K. These results highlight the coexistence of two distinct types of charge order and magnetism in LaRu_3_Si_2_ within the correlated Kagome lattice, along with superconductivity. This study sheds light on the intricate electronic and magnetic phenomena occurring in Kagome superconductors, providing valuable insights into their unique properties and potential applications.

## Graphical abstract

The coexistence of two distinct charge orders and emergent magnetism is uncovered in the correlated Kagome superconductor LaRu_3_Si_2_. A low‐temperature (1/6,0,0) charge order below 80 K induces magnetoresistance, and field‐driven magnetic response. An additional lower‐temperature scale marks time‐reversal symmetry breaking and Hall sign reversal, highlighting LaRu_3_Si_2_'s unique interplay of charge order with pronounced electronic responses, magnetism, and superconductivity. The calculations underscore the relevance of electronic correlations in this system.

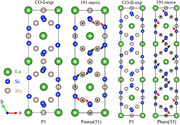

## 1 Introduction

Kagome superconductors,^[^ ^1^, ^2^, ^3^, ^4^, ^5^, ^6^, ^7^, ^8^, ^9^, ^10^, ^11^ ^]^ distinguished by their unique lattice structure resembling the traditional Japanese Kagome pattern, exhibit diverse electronic behaviors and have become a focal point in the study of condensed matter physics. An intriguing aspect is their propensity to host chiral charge order,^[^ ^12^, ^13^, ^14^, ^15^, ^16^, ^17^, ^18^ ^]^ a phenomenon where the electron density is spatially modulated to give the system a handedness, introducing unconventional electronic states. Understanding the interrelationship between Kagome superconductivity and chiral charge order is pivotal to harnessing their unique properties in the development of novel quantum materials and technologies.

Four distinct classes of Kagome lattice systems have recently been identified as exhibiting charge order: the *A*V_3_Sb_5_ family (where *A* = K, Rb, Cs),^[^ ^9^, ^10^, ^11^ ^]^ ScV_6_Sn_6_,^[^ ^19^, ^20^ ^]^ FeGe^[^ ^21^, ^22^ ^]^ and LaRu_3_Si_2_.^[^ ^5^ ^]^ In *A*V_3_Sb_5_, a correlated interplay of charge order emerges below *T*_co_ (around 80–110 K), along with a superconducting phase developing below *T*_c_ (approximately 1–3 K). ScV_6_Sn_6_, sharing a similar vanadium structural motif with *A*V_3_Sb_5_, displays charge order below *T*_co_ (around 90 K) but does not exhibit superconductivity down to the lowest measured temperatures. In contrast, FeGe, characterized as a correlated magnetic Kagome system, demonstrates an A‐type antiferromagnetic order below 400 K and a charge order below 100 K. One of the most remarkable features of the charged ordered state in all three materials is the emergence of possible time‐reversal symmetry (TRS) breaking, which features magnetic and electronic anomalies.^[^ ^12^, ^13^, ^14^, ^15^, ^16^, ^17^, ^23^, ^24^, ^25^, ^26^, ^27^, ^28^, ^29^, ^30^, ^31^, ^32^, ^33^, ^34^, ^35^, ^36^, ^37^, ^38^ ^]^ Theoretical considerations suggest that these features may be explained by a complex order parameter, realizing a higher angular‐momentum state, akin to unconventional superconducting orders. LaRu_3_Si_2_ stands out within the realm of bulk kagome‐lattice superconductors due to its exceptional characteristics. In particular, it has the highest superconducting critical temperature, reaching approximately 7 K. Furthermore, using single crystal X‐ray diffraction, we recently showed that LaRu_3_Si_2_ exhibits a charge‐ordered state at temperatures above room temperature under ambient conditions. The coexistence of both high‐temperature charge order and notable superconductivity makes LaRu_3_Si_2_ a particularly intriguing system for the exploration of quantum phenomena and potential applications in the field of condensed matter physics.

X‐ray diffraction measurements on LaRu_3_Si_2_ had previously^[^ ^5^ ^]^ been constrained to temperatures above 80 K, leaving uncertainties regarding the persistence of charge order into the superconducting state. Additionally, a comprehensive understanding of the electronic and magnetic response in the normal state remained elusive. Here, we address these questions to gain insights into the charge order across a broad temperature range, encompassing the superconducting state, as well as to elucidate the electronic and magnetic behaviors in the normal state. We employ a comprehensive set of techniques, including single‐crystal X‐ray diffraction, magnetotransport measurements, a combination of zero‐field and high‐field muon spin relaxation/rotation (µSR) experiments on a single crystal of LaRu_3_Si_2_, as well as Density Functional Theory (DFT) calculations. This multifaceted experimental and theoretical approach aims to provide a comprehensive understanding of the charge‐order phenomena and associated electronic and magnetic responses in both the normal and superconducting states of LaRu_3_Si_2_.

## 2 Results and Discussion

### 2.1 X‐Ray Diffraction Experiments and First Principles Calculations

**Figure 1:**
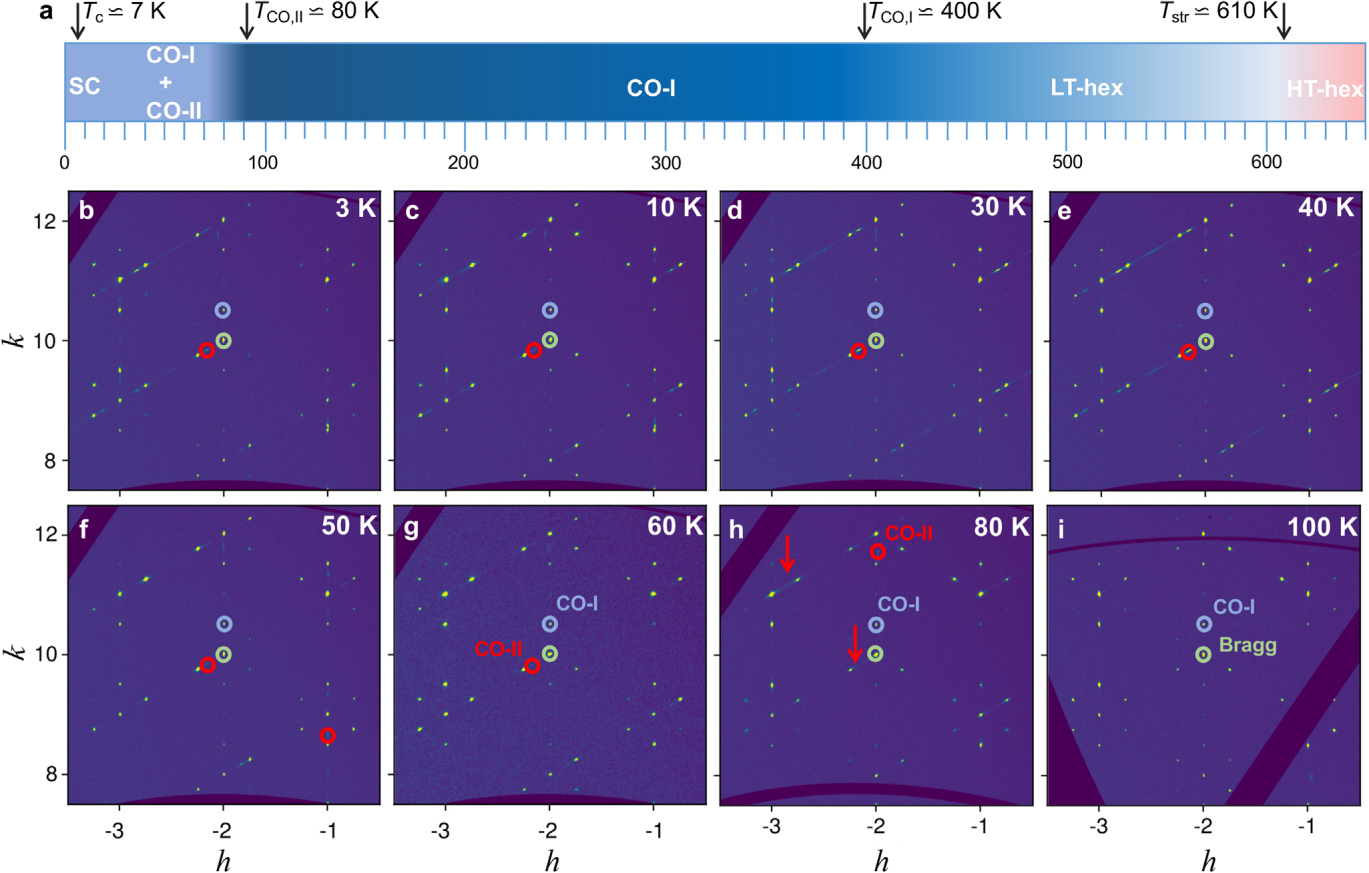
Charge order in LaRu_3_Si_2_. a) Schematic phase diagram as a function of temperature of LaRu_3_Si_2_. The arrows mark the structural phase transition temperature *T*_str_ from the high‐temperature hexagonal *P*6/*mmm* phase (SG No. 191) to the low‐temperature orthorhombic *Cccm* phase (SG No. 66), primary charge order transition temperature *T*_CO‐I_ and secondary charge order transition temperature *T*_CO‐II_. b–i) Reconstructed reciprocal space along the (h k 1) direction, performed at various temperatures above and below the superconducting transition temperature. Green, blue, and red circles mark the Bragg peak, primary charge order CO‐I peak, and secondary charge order CO‐II peak, respectively. Red arrows in panel (h) indicate the diffuse scattering at 1/6 periodicity, which is clustered into sharp diffraction spots at lower temperatures.

In **Figure** **1**b–i, we present reconstructed reciprocal‐space patterns along the (hk1) direction for various temperatures both above and below the superconducting critical temperature *T*_c_, which is approximately 7 K, for a single crystal of LaRu_3_Si_2_. At a higher temperature, *T* = 150 K, the diffraction pattern reveals fundamental Bragg peaks τ and superlattice peaks at *Q* = τ + *q*_*i*_ with *q*_1_ = ($\frac{1}{4}$,0,0) and *q*_2_ = ($0,\frac{1}{4}$,0). As the temperature decreases below *T*_co, 2_, approximately 80 K, an additional set of reflections emerges at positions corresponding to $q_{1}^{\prime }$=($\frac{1}{6},0,0$), $q_{2}^{\prime }$=(0, $\frac{1}{6},0$), and $q_{3}^{\prime }$=($\frac{1}{6},\frac{-1}{6},\;0$). Significantly, both $\frac{1}{4}$ and $\frac{1}{6}$ charge orders coexist below 80 K, persisting into the superconducting state. Figure 1a illustrates a schematic diagram showing the evolution of structural and electronic properties with respect to temperature. The diagram captures the progression of the material through a structural phase transition from the high‐temperature hexagonal *P*6/*mmm* phase (SG No. 191) to the low‐temperature orthorhombic *Cccm* phase (SG No. 66) at *T*_str_ ≃ 600 K. Subsequently, it highlights the onset of primary ($\frac{1}{4}$) charge order at *T*_co, 1_ ≃ 400 K and the emergence of secondary ($\frac{1}{6}$) charge order below *T*_co, 2_ ≃ 80 K. Notably, both charge orders coexist with superconductivity, manifesting below 7 K.

**Figure 2:**
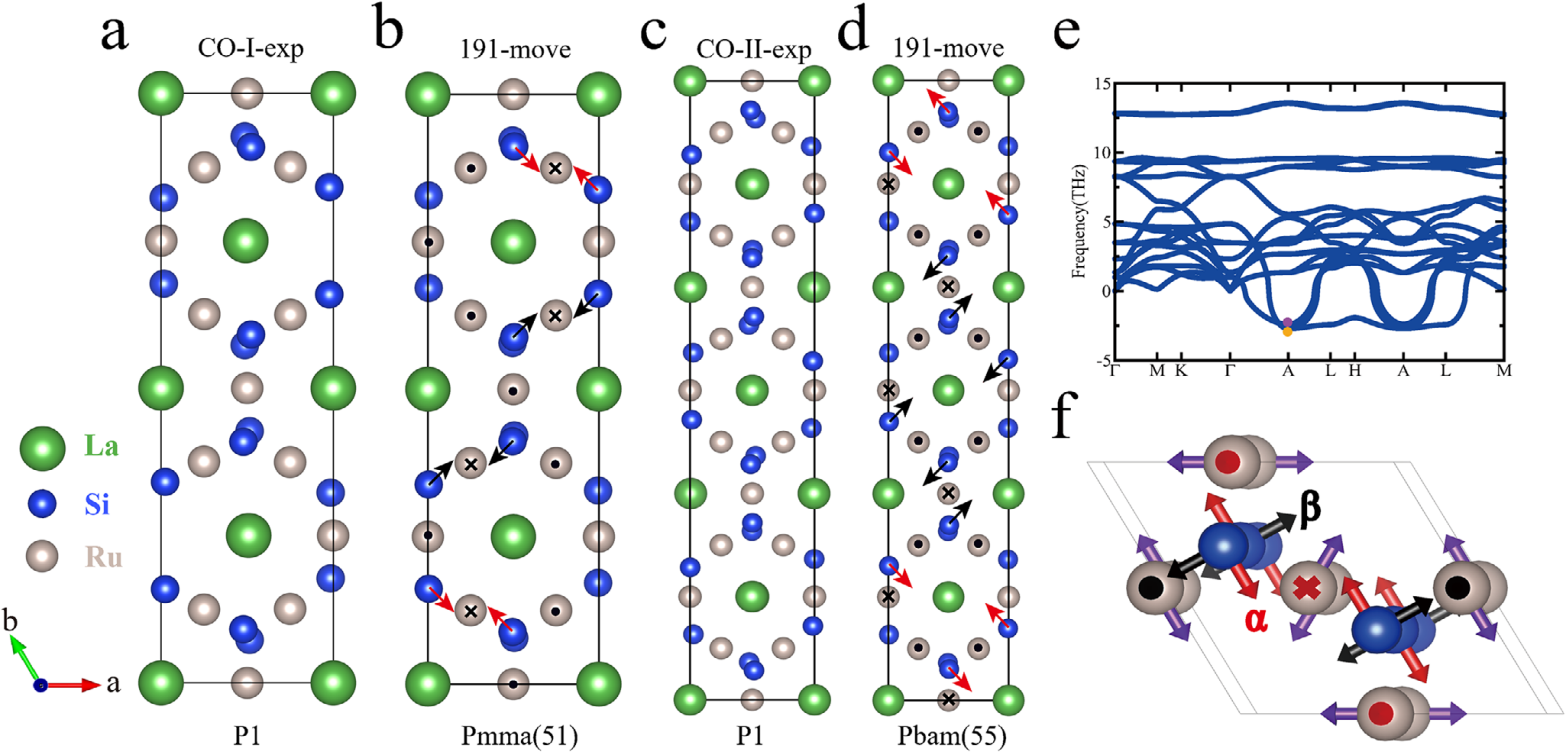
Different optimized structures of LaRu_3_Si_2_. a) Experimental determination of CO‐I structure. b) CO‐I (SG No. 51) is moved from the $a\times 2\sqrt{3}$×2c supercell of SG No. 191 structure. c) Experimental determination of CO‐II structure. d) Co‐II (SG No. 55) is moved from the $a\times 3\sqrt{3}$×2c supercell of SG No. 191 structure. The arrows represent in‐plane movements of Si atoms, the dots(crosses) represent upward (downward) movements of Ru atoms along c direction. e) Phonon spectrum of SG No. 191 structure with experimental lattice constants *a* = 5.688Å and *c* = 3.567Å. f) Movements of the atoms corresponding to the imaginary phonon modes of SG No. 191 structure. The purple (red and black) arrows correspond to the mode marked by purple (orange) dot in **e**. The red and black colors indicate α and β modes respectively.

To gain a deeper understanding of the charge orders in this system, we next present detailed results from first‐principles calculations of possible stable structures, the phonon spectrum, and the electronic structure. Previous studies have identified three structural phases for LaRu_3_Si_2_: the SG No. 191 structure at temperatures above T = 600 K and either the SG No. 193 or SG No. 176 structure in the range between 400 K and 600 K (Figures S1 and S2, Supporting Information). Notably, the optimized SG No. 176 structure consistently converges very closely to the SG No. 193 structure, a trend also observed in recent work by Wang et al.^[^ ^39^ ^]^ Building on experimental observations of the CO‐I and CO‐II phases, without imposing symmetry constraints (**Figure** **2**a,c),^[^ ^5^ ^]^ we construct supercells from the SG No. 191, SG No. 176, and SG No. 193 structures to derive the charge‐ordered (CO) phases by appropriately displacing atoms. Starting from the SG No. 191 structure, we generate $a\times 2\sqrt{3}$×2c and $a\times 3\sqrt{3}$×2c supercells, as shown in Figure 2b,d, respectively. In these figures, the arrows (dots and crosses) illustrate the displacement pattern of Si (Ru) atoms in the upper layer, while atoms in the lower layer move in the opposite direction. Upon structural optimization, the SG No. 51 and SG No. 55 phases emerge, corresponding to the CO‐I and CO‐II structures, respectively. These are henceforth denoted as CO‐I (SG No. 51) and CO‐II (SG No. 55). Applying the same methodology to the SG No. 176 and SG No. 193 structures results in the SG No. 11 and SG No. 2 structures for CO‐I, and the SG No. 14 and SG No. 2 structures for CO‐II, as depicted in Figure 9a– d. The optimized energies of these symmetry‐allowed CO‐I structures are comparable, with CO‐I (SG No. 51) being the most stable, as listed in Table 2. A similar trend is observed for the CO‐II phase, where CO‐II (SG No. 55) exhibits the lowest energy. These findings indicate that SG No. 191 serves as the parent structure for the CO‐I and CO‐II phases, rather than SG No. 176 or SG No. 193 (**Table** **1**).

**Table 1:** The energy of LaRu_3_Si_2_ for different fully optimized structures.

Structure	Energy (eV/f.u.)
191‐str (SG No. 191)	−47.16924
176‐str (SG No. 193)	−47.18212
193‐str (SG No. 193)	−47.18204
CO‐I (SG No. 51)	−47.25350
CO‐II (SG No. 55)	−47.25115

To investigate the origin of the CO‐I (SG No. 51) and CO‐II (SG No. 55) structures, we analyze the phonon spectrum of the SG No. 191 structure, as shown in Figure 2e. The spectrum reveals three imaginary modes in the *k*_*z*_ = π plane, with the minimum at the A(0,0,π) point. Among these, the two most unstable modes, labeled α and β, are degenerate, as indicated by the orange dot. These modes drive the movements of Si and Ru atoms, represented by arrows (dots and crosses) in red and black, respectively, in Figure 2f. By alternately locking these two modes along the b direction in the sequences ααββββαα and αααββββββααα (see the Si atoms in Figure 2b,d for clarity), the atomic displacements effectively lead to the formation of the CO‐I (SG No. 51) and CO‐II (SG No. 55) structures, respectively. Additionally, we observe that the second unstable mode, marked by a purple dot in Figure 2e, corresponds to the movement of Ru atoms, as indicated by the purple arrows in Figure 2f, leading to the formation of the SG No. 193 structure. These results confirm that the parent structure of CO‐I and CO‐II is SG No. 191 and that unstable phonon modes play an important role in the formation of these two charge‐ordered phases. Imaginary phonon modes may signal the role of electronic correlations, as shown in CuTe,^[^ ^40^ ^]^ highlighting the interplay between Coulomb interactions and electron‐phonon coupling. This is further supported by our detailed calculations of the phonon spectrum as a function of Hubbard U, which demonstrate that Coulomb repulsion within the DFT+U framework can significantly modify the imaginary phonon modes (see Figures S3 and S4, Supporting Information). Namely, the results highlight the crucial role of Coulomb repulsion in stabilizing the crystal structure of LaRu_3_Si_2_, emphasizing the importance of electronic correlations in this system. Within the DFT+U mean‐field framework, increasing U can suppress or eliminate the imaginary phonon modes—both serving as clear signatures of an important role played by Coulomb repulsion.

**Figure 3:**
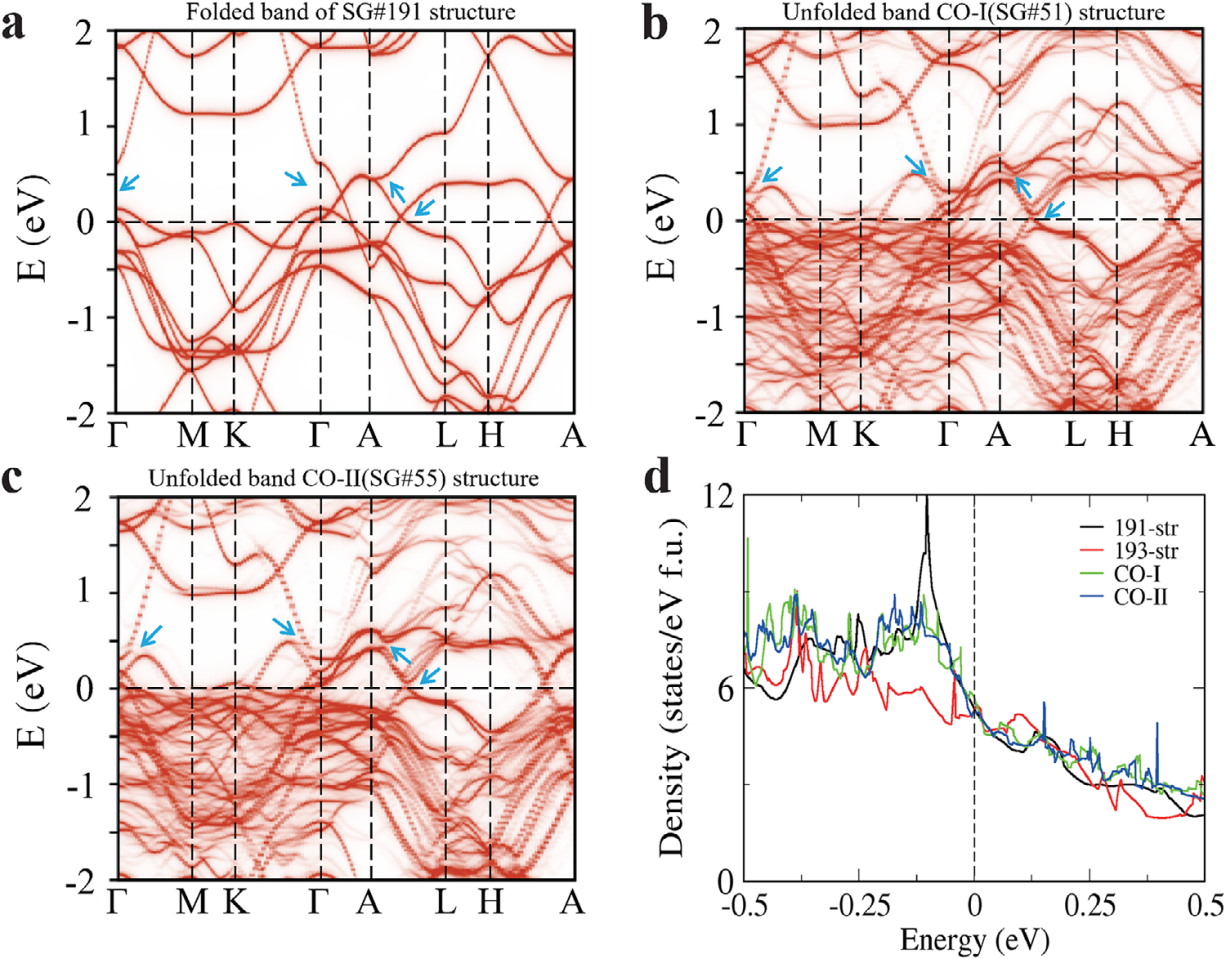
Calculated electronic structures for LaRu_3_Si_2_. a) The folded energy spectrum of SG No. 191 structure. b–c) The unfolded energy spectrum of CO‐I (SG No. 51) and CO‐II (SG No. 55) structures. The main difference of the spectrum near the Fermi level are pointed by blue arrows. d) The DOS of CO‐I, CO‐II, SG No. 191, and SG No. 193 structures.

We further calculate and compare the electronic spectra of the SG No. 191, CO‐I (SG No. 51), and CO‐II (SG No. 55) structures, as shown in **Figure** **3**a–c, respectively. In these figures, the spectra have been folded or unfolded into the Brillouin zone of the SG No. 191 structure. Compared to SG No. 191, the spectrum of CO structures brings the valence and conduction bands closer near the Γ point, lifts the degeneracy along the Γ‐A line, and opens the band crossing along the A‐L line, as indicated by the blue arrows. The total density of states (DOS) is calculated for the parent phase as well as for the CO‐I and CO‐II phases and is shown in Figure 3d. A suppression of narrow peak in the total DOS near the Fermi level is observed in the CO phases. Since the states at the Fermi level in LaRu_3_Si_2_ are primarily contributed by Ru‐4d electrons, the observed peak originates from these states. The suppression of this peak indicates that the transition into the charge‐ordered state has a significant impact on the density of states of Ru, which forms a Kagome layer. The interplay between unstable phonon modes, electronic structure modifications, and DOS suppression in the CO states suggests that the mechanism driving charge order in LaRu_3_Si_2_ involves both lattice distortions and electronic instabilities.

### 2.2 Resistivity and Magnetotransport Experiments

**Figure 4:**
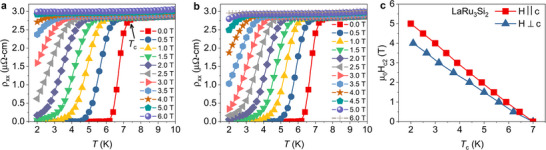
Weakly anisotropic superconducting properties of LaRu_3_Si_2_. The temperature dependence of the electrical resistivity of a single crystal of LaRu_3_Si_2_, measured under various magnetic fields applied perpendicular to the *c*‐axis (**a**) and parallel to the *c*‐axis (**b**). **c**) the magnetic field‐temperature phase diagram is derived from the data shown in panels **a** and **b**.

**Figure 5:**
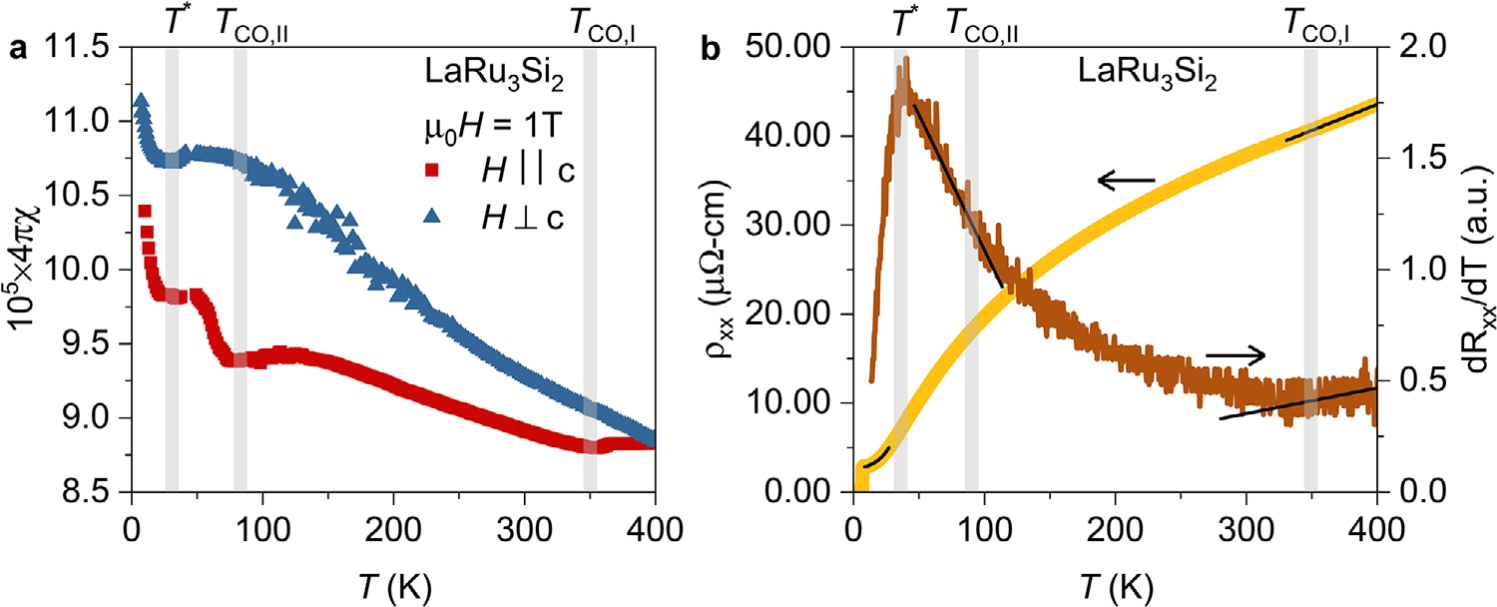
Magnetic and transport anomalies in LaRu_3_Si_2_. a) The temperature dependence of magnetic susceptibility, measured under the magnetic field of 1 T, applied parallel and perpendicular to *c*‐axis. b) Temperature dependence of the normal state longitudinal resistance ρ_*xx*_ and its first derivative. Vertical grey lines mark three characteristic temperatures T*, *T*_co, I_ and *T*_co, II_.

Next, we examined the superconducting transitions in LaRu_3_Si_2_ with the magnetic field applied both perpendicular (**Figure** **4**a) and parallel (Figure 4 **b**) to the *c* axis. The results indicate a weak anisotropy of the second critical field *H*_*c*2_. The estimated anisotropy of *H*_*c*2_ is approximately 1.25, a value significantly lower than the observed anisotropy of 8 in CsV_3_Sb_5_. In our investigation of the electronic properties of LaRu_3_Si_2_ in its normal state, comprehensive macroscopic magnetization and resistivity measurements were conducted under the influence of an applied magnetic field. **Figure** **5**a illustrates the temperature dependence of magnetic susceptibility at a magnetic field strength of 1 T, both parallel and perpendicular to the *c* axis. When the magnetic field is applied parallel to the *c* axis (*H* ∥ *c*), three distinct anomalies in the magnetic susceptibility are observed. Initially, a subtle drop in susceptibility occurs around 350 K, closely associated with the onset of the primary charge order at *T*_co, 1_. Subsequently, a sharp increase is evident below the secondary charge‐order temperature *T*_co, 2_, approximately 80 K, followed by an additional notable increase below T* ≈ 35 K. Interestingly, these magnetic susceptibility anomalies are prominently observed when the magnetic field is aligned along the *c* axis, but they are significantly less discernible when the field is applied in the kagome plane. In the case of *H* ⊥ *c*, only the transition at T* is evident. This indicates that the magnetic response across the charge‐order temperatures is anisotropic. The corresponding temperatures are highlighted in Figure 5b, where the temperature‐dependent resistivity under zero field, as well as its derivative, are presented. The derivative of the resistivity exhibits a peak at T*, while a subtle change in the slope of the resistivity curve is observed across the charge ordering temperatures *T*_co, 1_ and *T*_co, 2_.

**Figure 6:**
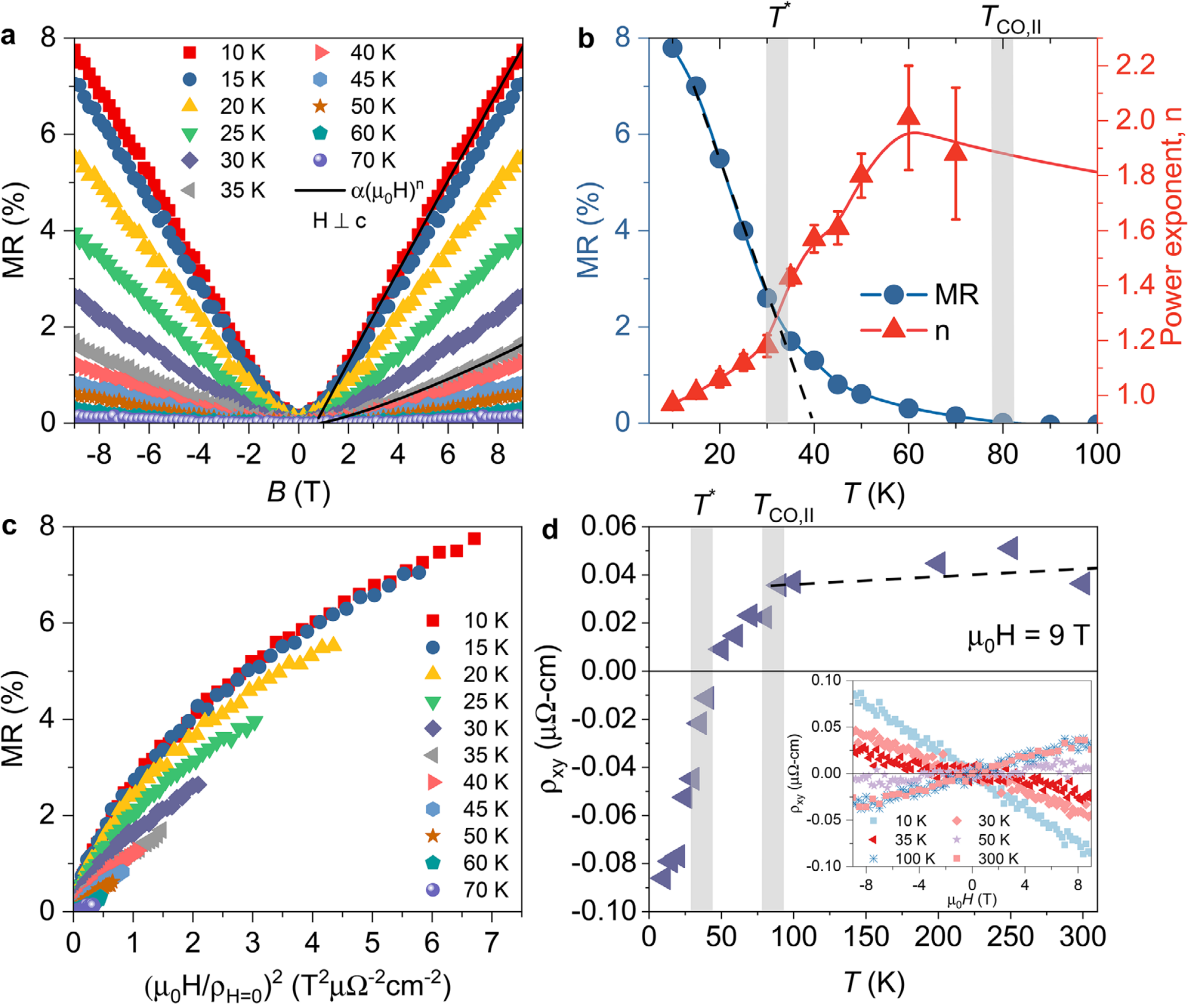
Magnetotransport characteristics for LaRu_3_Si_2_. a) The magnetoresistance measured at various temperatures across the charge ordering temperature *T*_co, 2_ ≃ 80 K. Black solid lines represent fits to the data by means of the following equation: Δρ/ρ_*H* = 0_ = α + β(µ_0_ *H*)^*n*^. b) The temperature dependence of the value of magnetoresistance at 9 T and the power exponent n. c) Kohler plot, Δρ/ρ_*H* = 0_ versus (µ_0_ *H*/ρ_*H* = 0_)^2^, of the magnetoresistance, plotted from field‐sweeps at various temperatures. d) The temperature dependence of the value of the Hall resistance ρ_*xy*_ at 9 T. Inset shows the Hall resistance, measured at various temperatures between 10 and 300 K.

The magnetotransport data obtained for LaRu_3_Si_2_ offer compelling evidence of the low‐temperature charge‐order transition. Magnetotransport,^[^ ^41^, ^42^, ^43^ ^]^ known for its sensitivity in detecting charge‐order transitions, utilizes magnetoresistance (MR) as a measure of the mean free path integrated over the Fermi surface.^[^ ^44^ ^]^ This method is particularly adept at detecting changes in scattering anisotropy and Fermi surface reconstructions. In **Figure** **6**a, we present the MR under a perpendicular magnetic field in LaRu_3_Si_2_ across the temperature range of 10 to 70 K. Within the primary $\frac{1}{4}$ charge‐ordered state, MR remains zero, only emerging below the $\frac{1}{6}$ charge ordering temperature *T*_co, 2_. The MR exhibits an initial increase at *T*_co, 2_, with a faster increase below T*, see Figure 6**b**. Initially following a standard quadratic dependence with µ_0_ *H* (applied magnetic field), MR can be well‐fitted to a polynomial: Δρ/ρ_*H* = 0_ = α + β(µ_0_ *H*)^*n*^, where α, β, and *n* are fitting parameters and *n* is close to 2. Yet, as the temperature lowers, *n* exhibits a decrease, with a notable change in slope across T*, and approaches *n* ≃ 1 well into the charge‐ordered state. This suggests that not only does the absolute value of MR alter with decreasing temperature, but the overall profile of MR also undergoes significant modification as the temperature decreases.

The Kohler plot, Δρ/ρ_*H* = 0_ versus (µ_0_ *H*/ρ*H* = 0)^2^, displayed in Figure 6c, is particularly noteworthy. The interesting observation in the Kohler plot is the singular behavior, where the MR data converge to a single line only below 15 K, in stark contrast to the pronounced temperature dependence evident at higher temperatures. Kohler's rule is a general principle stating that the normalized magnetoresistance, when plotted against a normalized magnetic field, should yield a universal curve for metals with simple Fermi surfaces. This principle tends to hold true even in materials with complex Fermi surfaces, provided there are no temperature‐dependent changes in the details of the Fermi surface. The deviation from the Kohler's rule in LaRu_3_Si_2_ suggests a fundamental alteration in the electronic structure and scattering processes associated with the onset of secondary charge order, emphasizing its significant impact on the transport properties of the material.

To further elucidate the electronic properties, Hall measurements were conducted, revealing a linear Hall resistivity at all temperatures. A linear Hall effect implies that transport is predominantly governed by a single type of charge carrier. In Figure 6d, the Hall effect exhibits a pronounced temperature‐dependence below *T*_co, 2_, with the sign of the Hall signal transitioning smoothly from positive (holes) to negative (electrons) across T*. These findings contribute valuable insights into the charge‐order‐induced alterations in the electronic structure of LaRu_3_Si_2_. The sign reversal observed across the charge‐ordering temperature in LaRu_3_Si_2_ parallels findings previously reported in 2H‐NbSe_2_^[^ ^45^ ^]^ and cuprate high‐temperature superconductors.^[^ ^46^, ^47^ ^]^ In those cases, this behavior was interpreted as a consequence of Fermi‐surface reconstruction associated with the onset of a density‐wave phase. It appears that in LaRu_3_Si_2_, the emergence of the secondary charge order similarly triggers a reconstruction of the Fermi surface, resulting in the appearance of a hole pocket. The similarities in the observed phenomena across different materials suggest a common underlying mechanism linked to charge order‐induced alterations in the electronic structure.

### 2.3 Muon Spin Spectroscopy

**Figure 7:**
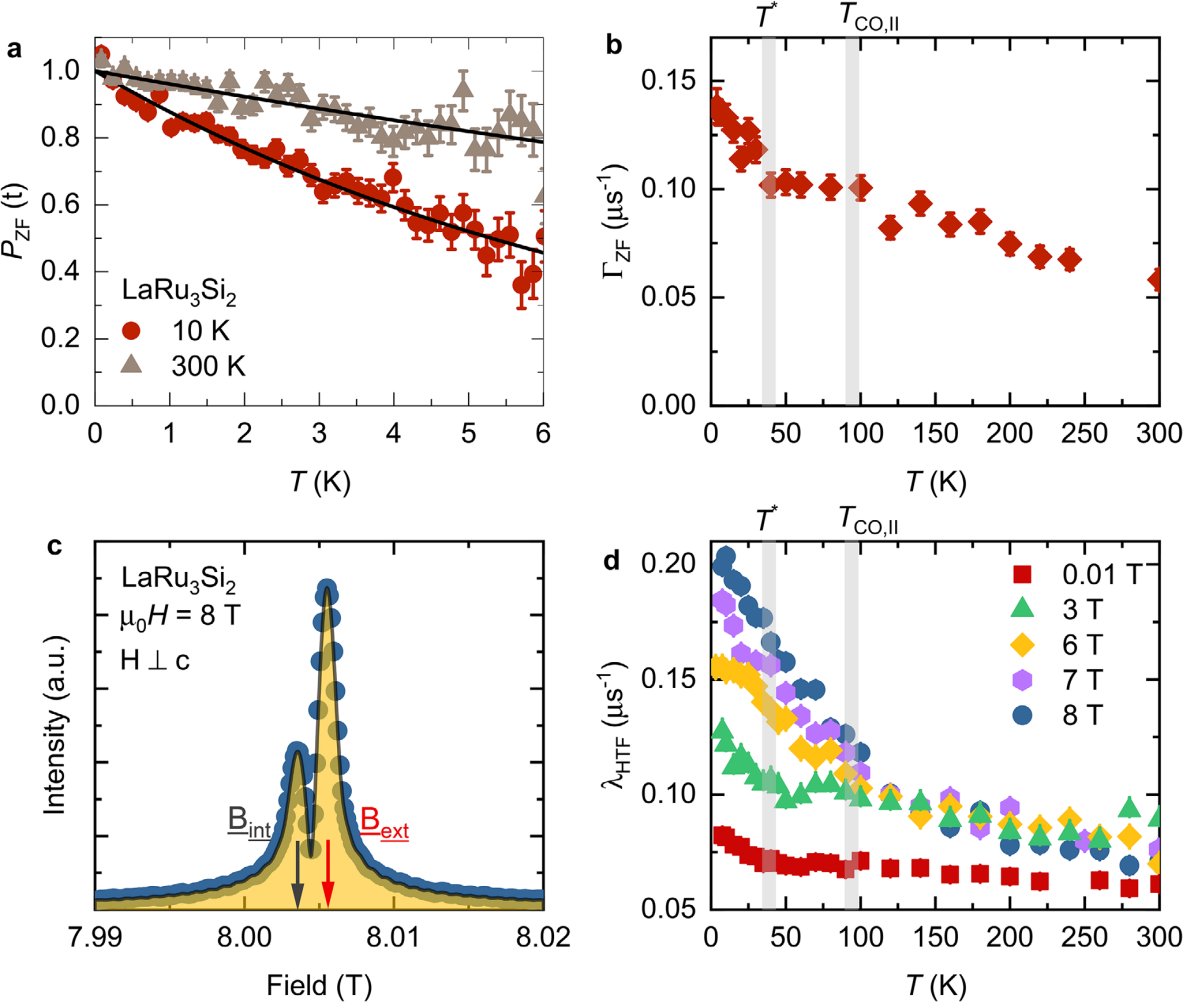
Magnetic response of the charge order in zero‐field and applying external magnetic fields in LaRu_3_Si_2_. a) The ZF µSR time spectra, obtained at *T* = 10 K and 300 K, all above the superconducting transition temperature *T*_c_. The solid black curves in panel **a** represent fits to the recorded time spectra, using the simple exponential function. Error bars are the standard error of the mean (s.e.m.) in about 10^6^ events. b) The temperature dependences of the zero‐field muon spin relaxation rate Γ_*ZF*_, obtained in a wide temperature range. The error bars represent the standard deviation of the fit parameters. c) Fourier transform of the µSR asymmetry spectra for the single crystal of LaRu_3_Si_2_ at 5 K in the presence of an applied field of µ_0_ *H* = 8T. The solid line is a two‐component signal fit. The peaks marked by the arrows denote the external and internal fields, determined as the mean values of the field distribution from the silver sample holder and from the sample, respectively. The short‐time‐window apodization function was used in the Fourier transform amplitude plot. d) Temperature dependence of the high transverse field muon spin relaxation rate λ_HTF_ for the single crystal of LaRu_3_Si_2_, measured under different *c*‐axis magnetic fields. The vertical grey line marks the transition temperature below which field effect was observed. The error bars represent the standard deviation of the fit parameters.

Subsequently, motivated by the significant magnetoresistance at low temperatures in LaRu_3_Si_2_ and the magnetic response reported in the charge‐ordered state of other Kagome‐lattice superconductors,^[^ ^13^, ^14^, ^23^, ^26^, ^27^ ^]^ we conducted zero‐field and high‐field µSR (muonspin rotation) experiments on a single crystalline sample of LaRu_3_Si_2_. The crystal, with dimensions 7 × 1.7 × 0.7 mm^3^, was affixed to a 5 mm circular silver sample holder using a small droplet of GE varnish during high‐field experiments. The zero‐field (*ZF*)‐µSR spectrum (**Figure** **7**a) is characterized by a weak depolarization of the muon spin ensemble, indicating no evidence of long‐range ordered magnetism in LaRu_3_Si_2_. However, it shows that the muon spin relaxation has a clearly observable temperature dependence. Since the full polarization can be recovered by the application of a small external longitudinal magnetic field, *B*_LF_ = 5 mT, the relaxation is, therefore, due to spontaneous fields which are static on the microsecond timescale. The zero‐field µSR spectra for LaRu_3_Si_2_ were fitted using the simple exponential function *P*_*ZF*_(*t*) = exp(−Γ_*ZF*_ *t*). Across the charge ordering temperature *T*_co, 2_ ≃ 80 K, there is only a change in the slope of Γ. However, a more significant observation occurs as the temperature is lowered below T* ≃ 35 K, where there is a notable increase in Γ (Figure 7b). Possible reason for the change of the relaxation rate across T* could be an additional modulation of the lattice structure that slightly alters the nuclear positions around the muon.^[^ ^48^ ^]^ However, a rough order of magnitude estimate yields that the structural distortions of the order of 0.1Å for the atoms closest to the muon would be needed to explain the observed effect in the second moment of the measured field distribution. This is a large effect that has not been seen by X‐ray diffraction experiments. Therefore, we can dismiss the structural distortion being the origin for the increase of the relaxation rate. Most importantly, our high‐field µSR results presented below definitively prove that there is indeed a strong contribution of electronic origin to the muon spin relaxation below the charge‐ordering temperature (see below). Therefore, we interpret our ZF‐µSR results as an indication that there is an enhanced width of internal fields sensed by the muon ensemble below T* ≃ 35 K. The increase in Γ below T* is estimated to be ≃ 0.035 µ*s* ^−1^, which can be interpreted as a characteristic field strength Γ/γ_µ_ ≃ 0.4 G.

To corroborate the zero‐field µSR results presented above, a comprehensive set of high‐field µSR experiments was conducted.^[^ ^49^ ^]^ Figure 7c illustrates the probability field distribution measured at 5 K in a magnetic field of 8 T applied perpendicular to the crystallographic *c* axis. Throughout the investigated temperature range, the µSR signals were characterized by two components: a signal with fast relaxation (λ_HTF_ ≃ 0.428(3) µ*s* ^−1^) and another with slower relaxation (0.05 µ*s* ^−1^). The fast relaxation signal, predominant in the µSR spectrum, arises from muons stopping within the sample and indicates a bulk response. Figure 7d depicts the behavior of the relaxation rate in a magnetic field of 0.01 T applied parallel to the *c* axis, resembling the temperature dependence observed in zero‐field conditions. At higher fields (3 T, 6 T, 7 T, and 8 T), the temperature dependence of the relaxation rate varies, with a clear and monotonous increase below the onset temperature *T*_co, 2_. This suggests that the low‐temperature relaxation rate in magnetic fields is predominantly influenced by electronic/magnetic contributions, as nuclear contributions are not susceptible to external field enhancement. The absolute increase of the relaxation rate between the onset of charge order *T*_co, 2_ and the base temperature in 8 T is Δλ_HTF_ ≃ 0.1 µ*s* ^−1^, which is smaller than in other kagome‐lattice superconductors such as KV_3_Sb_5_ and ScV_6_Sn_6_. Since the onset of the weak magnetic response aligns with the temperature *T*_co, 2_ of ($\frac{1}{6}$, 0, 0) charge order, this indicates a strong intertwinning between magnetic and charge channels. Additionally, a weak but non‐negligible field effect on the relaxation rate is observed above *T*_co, 2_, extending up to at least 300 K. This effect may be associated with the high‐temperature charge‐ordered state, and further experiments above 300 K are crucial for a more comprehensive understanding.

## 3 Summary and Conclusion

In this paper, we have presented three main findings: (1) Charge order with a propagation vector of ($\frac{1}{6}$, 0, 0) is identified in the single crystal of LaRu_3_Si_2_ below a critical temperature *T*_co, 2_ ≃ 80 K, coexisting with a previously reported room‐temperature primary charge order ($\frac{1}{4}$, 0, 0). The charge order persists into the superconducting state, highlighting the intricate interplay between superconductivity and distinct charge‐order phenomena. We provide insights into charge‐ordered structures, their influence on the electronic structure, and the DOS through Density Functional Theory (DFT) calculations. (2) Magnetotransport measurements reveal zero magnetoresistance within the primary charge‐ordered state and the appearance of magnetoresistance below *T*_co, 2_. The Kohler plot analysis, showing a violation of Kohler's rule, suggests a Fermi surface reconstruction induced by charge order. Anomalies in the Hall resistivity and its sign reversal across the charge‐ordering temperature further indicate a complex interplay of electronic states. The reconstruction of the Fermi surface is a fundamental aspect of such phenomena, wherein the charge order induces changes in the distribution of electronic states near the Fermi level. This restructuring can lead to anomalous transport properties, such as the observed temperature‐dependent Hall resistance and its sign reversal. By drawing parallels to observations in other materials such as cuprate high temperature superconductors, the study of LaRu_3_Si_2_ provides valuable insights into the universal aspects of charge order effects on electronic behavior, contributing to the broader understanding of quantum materials. (3) The µSR experiments conducted in both zero‐field and high‐field conditions have yielded noteworthy insights into the properties of LaRu_3_Si_2_. Below T*, approximately 35 K, there is a notable enhancement in the internal field width sensed by the muon ensemble. Additionally, a pronounced field‐induced enhancement of the relaxation rate is observed below *T*_co, 2_, which is around 80 K. Magnetic anomalies are also observed in bulk magnetization measurements under an applied field at *T*_co, 2_ and T*. These observations collectively indicate the presence of two distinct charge orders and magnetism coexisting with superconductivity in LaRu_3_Si_2_, making this system a rich platform for exploring various quantum states.

The interplay between unstable phonon modes, electronic structure modifications, and DOS suppression in the charge‐ordered (CO) states suggests that the mechanism driving charge order in LaRu_3_Si_2_ involves a combination of lattice distortions and electronic instabilities. It can be conjectured that the high‐temperature transition at *T*_co, 1_ is primarily phonon‐driven, while the low‐temperature transitions at *T*_co, 2_ and T* are governed by electronic interactions. This is supported by the strong electronic and magnetic anomalies observed across *T*_co, 2_ and T*, as reported in this work. Moreover, our recent^[^ ^50^ ^]^ high‐pressure magnetotransport and X‐ray diffraction studies reveal a clear correlation between superconductivity and the normal‐state electronic features associated with T* and *T*_co, 2_. This is evidenced by a striking positive relationship between *T*_c_ and several key phenomena: the strength of the resistivity anomaly at T*, a pressure‐induced crossover from long‐range to short‐range charge order below *T*_co, 2_, and the enhancement of magnetoresistance. The dome‐shaped pressure dependence of *T*_c_ appears to emerge from the intricate interplay between superconductivity, charge order, and these underlying electronic responses. These findings underscore the unconventional nature of superconductivity in LaRu_3_Si_2_, and highlight the importance of not just the presence of charge order, but also its spatial character and its coupling to the electronic and magnetic landscape of the normal state.

The debate about the origins of the various forms of charge order found in Kagome systems focuses on whether they are caused by electronic interactions or phononic effects. Electronically, there is significant interest in how sublattice interference and Q vectors connect the van Hove singularities (vHS).^[^ ^4^, ^32^ ^]^ The proximity of vHS to the Fermi level is key to the unusual superconductivity and 2×2 charge order in the *A*V_3_Sb_5_ (*A* = K, Rb, Cs) compounds.^[^ ^12^ ^]^ This 2×2 charge order pattern is also observed in the magnetic Kagome lattice of FeGe,^[^ ^21^, ^22^ ^]^ exhibiting similarities with the charge order found in Kagome superconductors *A*V_3_Sb_5_. In ScV_6_Sn_6_,^[^ ^19^ ^]^ the band structure also shows vHS near the Fermi level, but its charge order differs from the *A*V_3_Sb_5_ series. In ScV_6_Sn_6_,^[^ ^19^ ^]^ the primary driver for charge order is believed to be phonons, with the leading distortion involving an out‐of‐plane modulation of Sn and Sc sites.^[^ ^51^, ^52^, ^53^ ^]^ This is in contrast to the in‐plane Kagome breathing mode in *A*V_3_Sb_5_. The Kagome superconductor LaRu_3_Si_2_ also has vHS in its band structure, relating to Ru‐*dz* ^2^ orbitals near the Fermi level.^[^ ^8^ ^]^ Its charge order^[^ ^5^ ^]^ involves in‐plane displacement of Si atoms and out‐of‐plane displacements of some Ru atoms, differentiating it from both *A*V_3_Sb_5_ and ScV_6_Sn_6_. Additionally, the superconductor LaRu_3_Si_2_ exhibits a distinctive three‐dimensional character, both in its band structure and in its isotropic superconducting properties. This contrasts with the more two‐dimensional nature of the *A*V_3_Sb_5_. Despite these variations in charge‐order structures and their formation mechanisms (electronic and phononic) in different Kagome superconductors, they commonly exhibit indications of time‐reversal symmetry breaking and field‐induced enhancement of magnetism within the charge‐ordered state.^[^ ^13^, ^14^, ^20^, ^23^, ^26^ ^]^ This suggests a prevalent occurrence of magnetism in charge‐ordered Kagome lattices, tanscending specific fermiologies. LaRu_3_Si_2_ is unique among kagome superconductors due to the coexistence of multiple charge orders, each exhibiting distinct responses to magnetic fields, an additional temperature scale associated with time‐reversal symmetry breaking, and superconductivity. These findings deepen our understanding of how lattice and electronic effects interact to cause charge order instabilities in these complex systems. The presence of various orders at different temperatures might relate to orbitally selective ordering, adding complexity to the understanding of these materials' electronic and magnetic properties and highlighting LaRu_3_Si_2_s distinctiveness in the Kagome superconductor family.

## 4 Experimental Section

### X‐Ray Diffraction

X‐ray diffraction was performed at ID27, ESRF using monochromatic X‐rays with a wavelength of 0.3738 Å and a spotsize of 0.6x0.6 µ*m* ^2^. The sample was mounted on a membrane driven diamond anvil cell with 70 degrees angular aperture designed at ESRF. Helium was used as pressure transmitting medium and the applied pressure was measured by Ruby fluorescence. The diamond anvil cell was mounted into a Helium flow cryostat (ESRF). The applied pressure was kept constant to 0.3 GPa during cooling by adapting the membrane pressure. X‐ray diffraction was collected with a Eiger2 CdTe detector (DECTRIS AG, Baden‐Daettwil, Switzerland) in shutterless mode and continuous rotation over 64 degrees with readout every 0.1 degree. CrysAlisPro (Rigaku) was used for data reduction and TDS2EL (ESRF) for reciprocal space reconstructions.

### Magnetotransport

The magnetoresistance of the single‐crystal LaRu_3_Si_2_ was assessed using the physical property measurement system (PPMS‐9, Quantum Design) employing the conventional four‐probe technique. Four Pt‐wires (0.0254 mm diameter) were affixed to the single crystal using silver epoxy, forming a bar‐shaped specimen after polishing. A constant electrical current of 1 mA was applied, and the magnetic field was directed along the crystallographic *a*‐ and *c*‐axes, a configuration validated through Laue measurement.

### µSR Experiment

In a µSR (muon spin rotation) experiment, nearly 100% spin‐polarized muons µ^+^ are introduced into the sample one at a time. These positively charged µ^+^ particles thermally stabilize at interstitial lattice sites, effectively serving as magnetic microprobes within the material. In the presence of a magnetic field, the muon spin undergoes precession at the local field $B_{\mu }$ at the muon site, with a Larmor frequency $\nu_{\mu }$ given by $\gamma_{\mu }$/(2π)$B_{\mu }$, where $\gamma_{\mu }$/(2π) represents the muon gyromagnetic ratio and is equal to 135.5 MHz T^−1^.

Zero field (ZF) and transverse field (TF) µSR experiments on the single crystalline sample of LaRu_3_Si_2_ were performed on the GPS instrument and high‐field HAL‐9500 instrument, equipped with BlueFors vacuum‐loaded cryogen‐free dilution refrigerator (DR), at the Swiss Muon Source (SµS) at the Paul Scherrer Institut, in Villigen, Switzerland. Large single crystal piece was used for these measurements. The magnetic field was applied perpendicular to the crystallographic *c*‐axis. The crystal was mounted such that the *c*‐axis of it is perpendicular to the muon beam. Using the “spin rotator” at the πM3 beamline, muon spin was rotated (from its natural orientation, which is antiparallel to the momentum of the muon) by 44.5(3)° degrees with respect to the *c*‐axis of the crystal. So, the sample orientation is fixed but the muon spin was rotated. The rotation angle can be precisely determined to be 44.5(3)° by measurements in weak magnetic field, applied transverse to the muon spin polarization. Zero field and high transverse field µSR data analysis on single crystals of LaRu_3_Si_2_ were performed using both the so‐called asymmetry and single‐histogram modes.^[^ ^54^, ^55^ ^]^ The experimental data were analyzed using the MUSRFIT package.^[^ ^54^ ^]^

### First‐Principles Calculations

The first‐principles calculations are performed by the Vienna ab initio simulation package based on the density functional theory.^[^ ^56^, ^57^, ^58^ ^]^The generalized gradient approximation of Perdew, Burke, and Ernzerhof is used for the exchange correlation potential. The cut‐off energy for wave function expansion is 380 eV, and 9× 9 ×14 k‐point sampling grids are used in all calculations. The bulk structure of LaRu_3_Si_2_ is optimized until the force on each atom is less than 0.01 eV/Å. 2× 2 ×2 supercell is built to calculate the phonon dispersion by using the density functional perturbation theory and Phonopy.^[^ ^39^ ^]^

## Conflict of Interest

The authors declare no conflict of interest.

## Author Contributions

Z.G. initiated the study, designed the research plan and supervised the project. C.M.III, V.S., and M.Y. contributed equally to the paper. Crystal growth: H.N. and S.N. Magnetotransport experiments: V.S., M.B., and Z.G. Magnetization experiments: C.M.III., M.M., and Z.G. X‐ray diffraction experiments, analysis and corresponding discussions: B.W., C.M.III, I.P., V.S., G.G., J.N.G., S.S., D.J.G., and Z.G. DFT calculations and corresponding discussions: M.Y., G.X., and Z.G. µSR experiments and corresponding discussions: C.M.III., V.S., R.K., H.L., T.N., M.H.F., M.Z.H., J.‐X.Y., and Z.G. Data analysis and figure development: Z.G., C.M.III., V.S., B.W., M.Y. Writing of the paper: Z.G. with contributions from all authors. All authors discussed the results, interpretation, and conclusion.

## Supporting information

Supporting Information

## Data Availability

The X‐ray diffraction data is available at https://doi.org/10.15151/ESRF‐ES‐1402335301. The rest of the results are available from corresponding authors upon request.
