# Supplementary material for: Coexisting Multiple Charge Orders and Magnetism in the Kagome Superconductor LaRu3Si2
Source: Adv Mater. 2025 Jul 23;37(40):2503065. doi: 10.1002/adma.202503065 (PMC12510282; doi:10.1002/adma.202503065)
Supplement: Supplementary file 1 — Supporting Information [file ADMA-37-2503065-s001.pdf]

ADVANCED MATERIALS

Supporting Information

for *Adv. Mater.*, DOI 10.1002/adma.202503065

Coexisting Multiple Charge Orders and Magnetism in the Kagome Superconductor $LaRu_3Si_2$

C. Mielke III, V. Sazgari, I. Plokhikh, Mingsheng Yi, S. Shin, H. Nakamura, J.N. Graham, J. Küspert, I. Biało, G. Garbarino, D. Das, M. Medarde, M. Bartkowiak, J.-X. Yin, S.S. Islam, R. Khasanov, H. Luetkens, M.Z. Hasan, E. Pomjakushina, M. H. Fischer, J. Chang, T. Neupert, S. Nakatsuji, B. Wehinger, Gang Xu, D.J. Gawryluk* and Z. Guguchia**

Supporting Information

Coexisting Multiple Charge Orders and Magnetism in the Kagome Superconductor $LaRu_3Si_2$

C. Mielke III,[1,2,*] V. Sazgari,[1,*] I. Plokhikh,[1] Mingsheng Yi,[3,*] S. Shin,[1] H. Nakamura,[4] J. Graham,[1] J. Küspert,[2] I. Biało,[2] G. Garbarino,[5] D. Das,[1] M. Medarde,[1] M. Bartkowiak,[1] J.-X. Yin,[6] S.S. Islam,[1] R. Khasanov,[1] H. Luetkens,[1] M.Z. Hasan,[7] E. Pomjakushina,[1] M. H. Fischer,[2] J. Chang,[2] T. Neupert,[2] S. Nakatsuji,[4] B. Wehinger,[5] Gang Xu,[3,†] D.J. Gawryluk,[1,‡] and Z. Guguchia[1,§]

[1]*PSI Center for Neutron and Muon Sciences CNM, 5232 Villigen PSI, Switzerland*
[2]*Physik-Institut, Universität Zürich, Winterthurerstrasse 190, CH-8057 Zürich, Switzerland*
[3]*Wuhan National High Magnetic Field Center and School of Physics, Huazhong University of Science and Technology, Wuhan 430074, China*
[4]*Institute for Solid State Physics (ISSP), University of Tokyo, Kashiwa, Chiba 277-8581, Japan*
[5]*European Synchrotron Radiation Facility, 71 Avenue des Martyrs, 38000 Grenoble, France*
[6]*Department of Physics, Southern University of Science and Technology, Shenzhen, Guangdong, 518055, China*
[7]*Laboratory for Topological Quantum Matter and Advanced Spectroscopy (B7), Department of Physics, Princeton University, Princeton, New Jersey 08544, USA*

Supplementary Figures S1a-c show the SG No. 191, SG No. 193 and SG No. 176 structures of $LaRu_3Si_2$, in which the Kagome layer of Ru in SG No. 193 and SG No. 176 structures are distorted and doubled along c axis. In order to determine the structure of the CO-I phase (CO-II phase), we construct the a$\times 2\sqrt{3}\times$2c (a$\times 3\sqrt{3}\times$2c) supercell from the SG No. 191 structure as described in the main text, and the a$\times 2\sqrt{3}\times$c (a$\times 3\sqrt{3}\times$c) supercell from the SG No. 176 and SG No. 193 structures as shown in the Supplementary Figures S2a,b (Supplementary Figures S2c,d). By comparing these supercell structures with the experimental observation of CO structures without considering the symmetry, we find that the CO structures can be obtained by moving the atoms accordingly (see the arrows, dots and crosses in the Supplementary Figures S2a-d), in which the arrows (dots and crosses) only show the manner of moving the Si (Ru) atoms in the upper layer while the atoms in the lower layer move in the opposite direction. After optimization, the SG No. 2 and SG No. 11 structures are obtained for CO-I, SG No. 2 and SG No. 14 structures are obtained for CO-II, corresponding to the Supplementary Figures S2a-d respectively.

Supplementary Figures S2e shows the phonon spectrum of the SG No. 176 structure (the same for SG No. 193 structure), which presents two imaginary modes in the $k_z = 0$ plane with the minimum at the Γ(0,0,0) point. Comparing the phonon spectrum of SG No. 191 and SG No. 193, we find that the second unstable mode of SG No. 191 (the purple dot in Supplementary Figures S2e) disappears in SG No. 193, suggesting that this mode is the inducement to transform SG No. 191 into SG No. 193. The unstable two modes, called α and β, are degenerate as marked by black dot in the Supplementary Figures S2e, corresponding to the movements of the Si (Ru) atoms illustrated by red and black arrows (dots and crosses) in the Supplementary Figure S2f, respectively. Alternatively locking these two modes along the b direction in $\alpha\alpha\beta\beta\beta\beta\alpha\alpha$ and $\alpha\alpha\alpha\beta\beta\beta\beta\beta\beta\alpha\alpha\alpha$ manners can effectively lead to atomic motions in the structures of the Supplementary Figures S2b-d, respectively.

We have performed the phonon calculations based on the fully optimized SG No. 191 structure by both PBE potential and PBE + U =1 eV method, respectively. As shown in Figure. S3, it reveals that PBE optimized structure

CO-latt ion optimization	Energy (eV/f.u.)
CO-I (SG No. 51)	-47.24544
CO-I (SG No. 2)	-47.24534
CO-I (SG No. 11)	-47.24536
CO-II (SG No. 55)	-47.24095
CO-II (SG No. 2)	-47.24099
CO-II (SG No. 14)	-47.24097

TABLE S1. **Energy of CO-I and CO-II with different symmetries after ion optimization.**

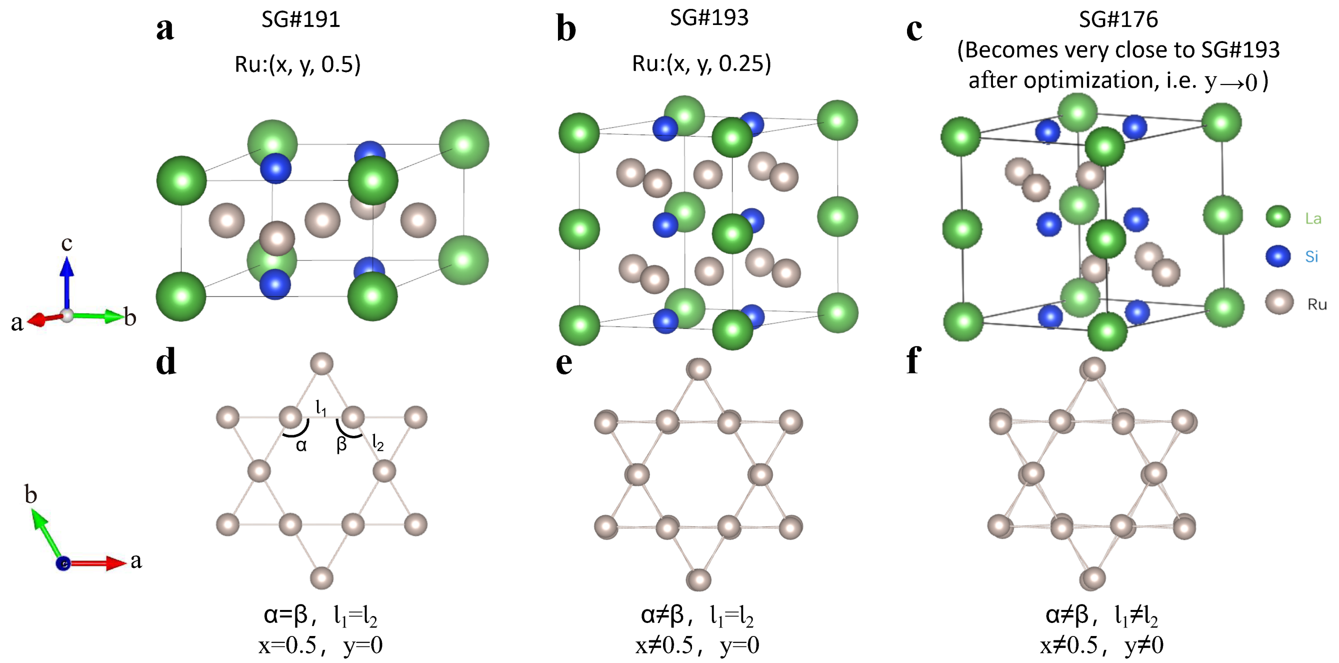

FIG. S1. **Possible crystal structures for $LaRu_3Si_2$. a − c**, Three possible crystal structures of $LaRu_3Si_2$ are SG No. 191, SG No. 193 and SG No. 176 structures. **d − f**, Top view of the Ru plane. The angles α, β and side lengths l_1, l_2 of the hexagon, the fractional coordinates of Ru atoms x,y, and the space group are shown, respectively.

is even worse, which leads to more imaginary phonon modes compared to the results in Fig. 1e. In contrast, it seems that the PBE + U =1 eV method could suppress the imaginary phonon modes (see Figure. S3b). However, we would like to emphasize that the lattice constant $c = 3.2936$ Å obtained by the PBE + U =1 eV method is obviously unreasonable, which is reduced more than 7.6 % compared to the experimental c = 3.567 Å. Such results are clearly contrary to the physical fact that Coulomb repulsion U usually leads to an enhancement of the lattice constant. It seems that the mean field approach adopted in DFT+U is not a good method to deal with the correlation effect in $LaRu_3Si_2$. Therefore, a more accurate approach to deal with electron-electron correlation and also the investigation including electron-phonon interaction is warranted in the future. Moreover, our calculations show that the small c is beneficial for the suppression of the imaginary phonon modes, while large c usually gives rise to the enhancement of the imaginary phonon modes as shown in Figure S4. All these results indicate that Coulomb repulsion U plays a crucial role in stabilizing the crystal structure of $LaRu_3Si_2$, underscoring the importance of electronic correlations in this system. Within the DFT+U mean-field framework, increasing U can suppress or eliminate the imaginary phonon modes, but this also leads to an unphysical expansion of the lattice constant c, deviating even further from the experimental value.

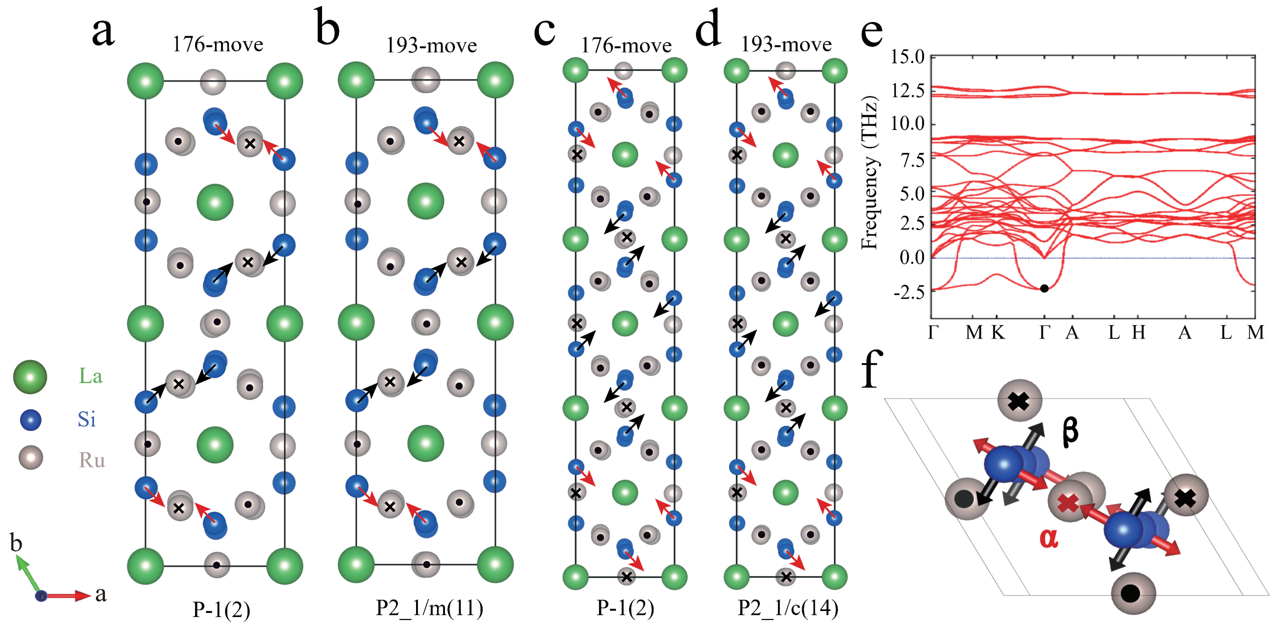

FIG. S2. **Atomic displacements in the charge ordered states in $LaRu_3Si_2$.** **a**, CO-I (SG No. 2) is moved from the a×2$\sqrt{3}$×2c supercell of SG No. 176 structure. **b**, CO-I (SG No 11) is moved from the a×2$\sqrt{3}$×2c supercell of SG No. 193 structure. **c**, CO-II (SG No. 2) is moved from the a×3$\sqrt{3}$×2c supercell of SG No. 176 structure. **d**, CO-II (SG No. 14) is moved from the a×3$\sqrt{3}$×2c supercell of SG No. 193 structure. The arrows represent in-plane movements of Si atoms, the dots (crosses) represent upward (downward) movements of Ru atoms along c direction. **e**, Phonon spectrum of SG No. 176 structure. **f**, Movements of the atoms corresponding to the imaginary phonon modes of SG No. 176 structure. The red and black arrows correspond to the mode marked by black dot in **e**. The red and black modes are represented by α and β respectively.

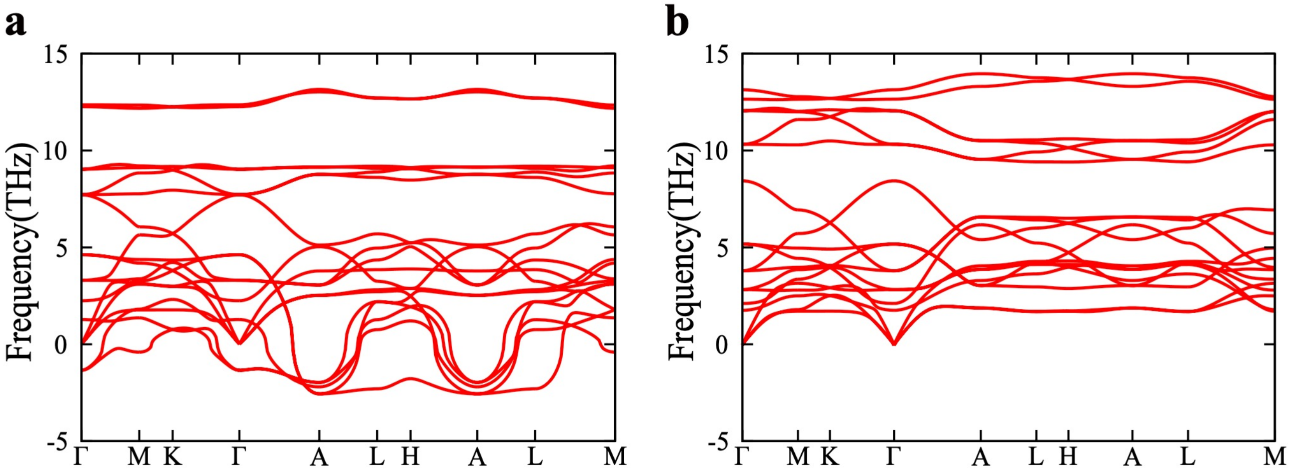

FIG. S3. **Phonon spectrum based on the optimized SG No. 191 structures.** **a**, Phonon spectrum calculated by the PBE potential with optimized lattice constants a = 5.7095 Å and c = 3.5805 Å. **a**, Phonon spectrum calculated by PBE+U = 1eV method with optimized lattice constants a = 5.9381 Å and c = 3.2936 Å.

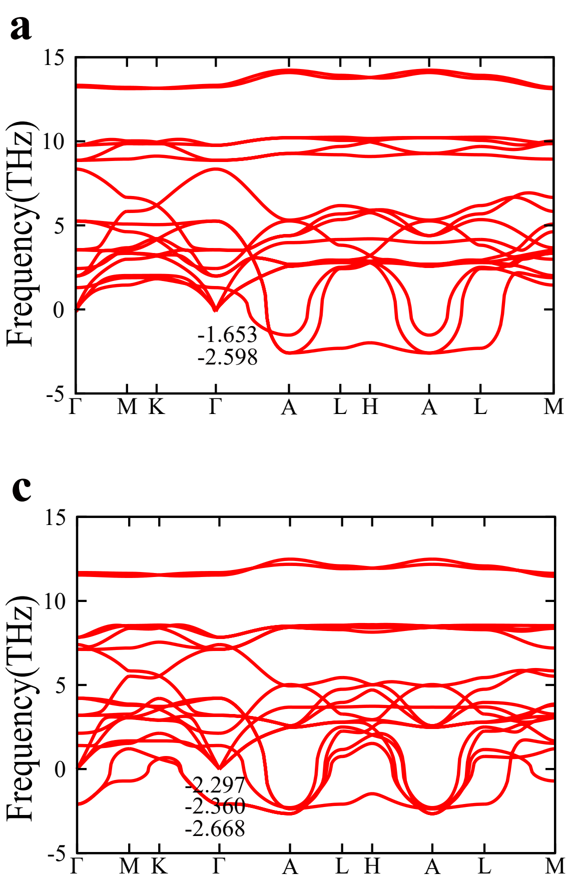

FIG. S4. **Phonon spectrum calculated by PBE potential for various lattice constants.** Phonon spectrum calculated by PBE potential with experimental in-plane lattice constant a = 5.688Å but different c, such as 3 % reduction, i.e., c = 3. 4731 Å (a), 2 % reduction, i.e., c = 3.5089 Å (b), 2 % enlargement, i.e., c = 3.6521 Å (c), and 3 % enlargement, i.e., c = 3.6879 Å (d).
